# Can we establish a hierarchy among trastuzumab biosimilar candidates?

**DOI:** 10.1038/s41416-018-0171-1

**Published:** 2018-07-13

**Authors:** Xavier Pivot, Thierry Petit

**Affiliations:** Porte de l’Hopital Strasbourg, Centre Paul Strauss, Strasbourg, France

## Abstract

The European patent for intravenous trastuzumab lapsed in 2017, and this stimulated research into a number of trastuzumab biosimilars. Quality assessment of their development and clinical results might enable establishment of a clinical hierarchy of these agents. This editorial will underline the key points for consideration when determining such an evaluation.

## Main

The extraordinary clinical achievements of trastuzumab have made history in the systemic management of breast cancer. Unfortunately, it is not uniformly available for routine use owing to its prohibitively high cost. With financial contingencies following the economic crisis together with rapidly increasing healthcare costs, even the richest countries are exploring ways to reduce their healthcare expenditures. In 2017, the patent for intravenous trastuzumab (Herceptin) expired across Europe, which stimulated the development of numerous trastuzumab biosimilar agents (Table 1).^1–5^ In this issue of the *British Journal of Cancer*, Lammers et al. report evidence establishing another step towards the registration of PF-05280014, a trastuzumab biosimilar candidate developed by Pfizer.^1,6–8^ This significant trial has provided clinical efficacy results of this candidate in patients with early breast cancer, and insight into its pharmacokinetic (PK) non-inferiority.

**Table 1:** Results of the randomised clinical studies achieved by major trastuzumab biosimilar candidates.^1–5,8^

	Amgen ABP980^5^	Samsung BioEpis/Merck SB3^2^	Celltrion CT-P6^4a^	Pfizer PF-05280014^1,8^	Biocon/Mylan MYL-1401O^3^
Neoadjuvant setting	✓	✓	✓	✓	
*N*	725	875	549	226	
Metastastic setting	—	—	✓	✓	✓
*N*			475	707	458
Primary endpoint	Total pCR	Breast pCR	EBC: total pCR MBC: ORR	EBC: PK (C_trough_ > 20 μg/ml at Cycle 5 [Cycle 6 predose]) MBC: ORR	ORR
Equivalence margins for efficacy (risk difference)	90% CI ± 13%	95% CI ± 13%	EBC: 95% CI ± 15% MBC: 95% CI ± 15%	MBC: 95% CI 0.8–1.25 (risk ratio)	95% CI ± 15%
Primary endpoint (Biosimilar vs Herceptin)	48% vs 40.5%	51.7% vs 42%	EBC 46.8% vs 50.4%	MBC 62.5% vs 66.5%	69.6% vs 64%
Results observed (risk difference)	7.3% (1.2%,13.4%)	10.7% (4.1%, 17.3%)	EBC: −4% (12%, 5%)	MBC (risk ratio): 0.94 (0.84–1.05)	5.53% (−3.08, 14.04%)

*CI* confidence interval, *C*_*trough*_ plasma concentration, *ORR* objective response rate, *pCR* pathological complete response, *EBC* early breast cancer; *MBC* metastatic breast cancer, *PK* pharmacokinetic.

^a^Metastatic assessment did not support the registration of CPT6

The development of a biosimilar drug requires the collation of extensive pre-clinical comparability studies to demonstrate similar structural, physicochemical, and functional biological characteristics with the referent medical product.^9,10^. PK comparability in animal models is required prior to the first in-human study, and this is usually aimed at demonstrating PK equivalence between the biosimilar candidate and the referent. These steps have already been successfully achieved for PF-05280014.^6,7^ Treatment with trastuzumab can result in the production of anti-trastuzumab antibodies; therefore, initial phase I trials for PK assessment include healthy male subjects, because they are less likely to require treatment with trastuzumab for breast cancer in the future. Notably, several previous studies have validated the absence of observable discrepancies in PK profiles for trastuzumab between healthy volunteers and patients.^2,4,11,12^ Iterative drug administration causes an accumulation of plasma drug levels, thus resulting in an increase of the average AUC_(0-t)_. For trastuzumab exposure, large variability in the AUC_(0-t)_ is observed up to cycle 5, beyond which the values become stable and homogeneous over time.^13,14^ Therefore, a large randomised study aimed at comparing the activities should also perform a PK assessment in patients receiving iterative administration.

A randomised clinical study represents the ultimate step in the path towards drug registration, with the intention of providing evidence for similar efficacy between the biosimilar candidate and the reference medical product in a sensitive population.^10^ The strength of the statistical demonstration of equivalence is a key parameter in the quality of this comparability exercise.^15^ The approach recommended by regulatory authorities for deriving equivalence margins relies on preserving the reference treatment effect, estimated using a meta-analysis focusing on major studies. These recommendations posit a 50–60% preservation of the reference treatment effect from the average effect, or from the lower boundary of the 95% confidence interval. Variable margins of equivalence using different ranges of confidence were pre-specified to define equivalence (Table 1).^1–5^ This variability reflects the difficulty in reaching a consensus on the acceptable and/or reasonable difference in efficacy of a biosimilar compound.

The second parameter of interest to consider is the population studied and the endpoint criterion used to claim equivalence. According to guidelines, clinical trials should be carried out on a sensitive and homogenous patient population, using endpoints that will; most easily detect differences between the biosimilar and the reference product.^10^ Archetypical survival endpoints dictate prolonged follow-up with associated increased development costs;these are in contradiction with biosimilar development strategies. Two population options are available: comparison at the metastatic setting based on objective response rate (ORR), or assessment at neoadjuvant setting using pathological complete response (pCR). The neoadjuvant setting is a more homogeneous population, meaning fewer uncertainty factors. In contrast, efficacy at the metastatic setting could be impacted by previous treatment exposures, including chemotherapy and anti-HER2-targeted agents, and by tumour burden or the type of metastatic sites involved. The neoadjuvant setting selects patients with localised disease who are naïve to any anticancer therapy. Moreover, the neoadjuvant setting allows the use of pCR, which is a better efficacy assessment criterion and is related to survival outcome.^16–18^ ORR for metastatic lesions is not related to survival outcomes, and has not been used for the basis of any drug registration in breast cancer.^19^ Considering these points, the neoadjuvant setting is considered to be an important area for development in HER2-positive breast cancer.^20^ If a hierarchy based on clinical assessment could be developed for trastuzumab biosimilar candidates, then the agents with a favourable clinical assessment using pCR after neoadjuvant therapy might appear at the top of such classification (Table 1).^1–5^

A single clinical study, in either the metastatic or the neoadjuvant setting, is usually sufficient to prove the equivalent efficacy of a biosimilar drug. CPT-6 was previously assessed in studies using both settings, but this was due to small changes in the production process that required duplication of all the comparability exercises .^4^ For PF-05280014, clinical equivalence was based on a study in the metastatic setting, which was reported at ESMO by Pegram et al.^1^ Lammers et al. report on a second trial,^8^ innovatively added into the development, which has also assessed PF-05280014 in the neoadjuvant setting. Paired with their earlier findings, this second trial may appear as the icing on the cake. In addition, the authors performed a PK analysis in patients receiving iterative cycles of trastuzumab, an area that was definitley needed, as previously underlined. An assessment of trough plasma levels (C_trough_) at cycle 5 is relevant, because a key PK parameter to consider is a C_trough_ value higher than the threshold of biological activity. Whereas investigators of other biosimilar competitors have studied this PK in an ancillary sub-study of a unique randomised clinical trial,^2–4^ the development of PF-05280014 has now involved a dedicated randomised trial to evaluate PK non-inferiority with Herceptin.

The neoadjuvant trial reported in the study by Lammers et al. has provided supplementary clinical data demonstrating comparable efficacy, based on pCR results.^8^ The 47% and 50% pCR rates achieved by PF-05280014 and Herceptin, respectively, provide a stratified difference for pCR of –2.81% (95% CI –16.58%/10.96%) based on investigator assessment. These differences and the 95% CI values are in line with the reported values provided by other biosimilar competitors in the same setting (Table 1).^2,3,5^ Thus, our neoadjuvant-based pCR assessment demonstrates comparable efficacy and provides complementary data supporting the success of this biosimilar candidate in the competition for market access.

Several trastuzumab biosimilar candidates have already obtained authorisation for routine use in Europe, and a few of them might be in use within months. The next step will be to assess the impact in terms of cost savings resulting from the implementation of these biosimilars in these countries. To this day, debate over the cost decrease prospects for trastuzumab with the use of biosimilars is still at a speculative stage; further developments in the ongoing year are awaited with great interest.
